# A Case of an Vertebral Artery Dissecting Aneurysm Involving the Posterior Inferior Cerebellar Artery Treated With Aneurysmal Embolization and Proximal Parent Artery Occlusion Under Balloon Protection via the Contralateral Vertebral Artery

**DOI:** 10.7759/cureus.85650

**Published:** 2025-06-09

**Authors:** Takuya Nakamura, Yoshiki Hanaoka, Jun-Ichi Koyama, Takahiro Murata, Masafumi Kuroiwa, Tetsuyoshi Horiuchi

**Affiliations:** 1 Neurosurgery, Shinshu University School of Medicine, Matsumoto, JPN; 2 Neurosurgery, Ina Central Hospital, Ina, JPN; 3 Neurosurgery, Kobayashi Neurosurgical Hospital, Nagano, JPN; 4 Neurosurgery, Minaminagano Medical Center, Shinonoi General Hospital, Nagano, JPN

**Keywords:** balloon assist, coil embolization, posterior inferior cerebellar artery, subarachnoid hemorrhage, vertebral artery dissection

## Abstract

We experienced a case of subarachnoid hemorrhage due to a rupture of a vertebral artery dissecting aneurysm (VADA) involving the posterior inferior cerebellar artery (PICA) treated with a novel endovascular technique, which used a balloon catheter to protect the PICA origin via the contralateral vertebral artery. We performed coil embolization of the VADA and proximal parent artery occlusion via the ipsilateral vertebral artery under protection of the PICA. Using a balloon catheter instead of a stent, dense packing was achieved while preserving the PICA and avoiding hemorrhagic complications with stent-related antiplatelet therapy. Although further assessment is needed to clarify the potential risks and limitations, this technique can be a useful treatment option for VADA involving the PICA.

## Introduction

A ruptured vertebral artery (VA) dissecting aneurysm (VADA) requires emergent treatment due to life-threatening rebleeding [1]. Endovascular procedures are categorized into two methods: the reconstructive method, which preserves the parent artery using stents, and the deconstructive method, which involves occlusion of the parent artery [2]. The reconstructive method has the advantage of maintaining antegrade flow in the VA and posterior inferior cerebellar artery (PICA); however, it carries the risk of aneurysmal regrowth and rebleeding due to residual blood flow. Furthermore, the risk of early rebleeding associated with stent-related antiplatelet therapy should be considered [3]. In contrast, the deconstructive method provides definitive embolization of the VADA, but it poses the risk of ischemic complications of the PICA and anterior spinal artery (ASA) territories. Although proximal parent artery occlusion (PAO) can reduce the hemodynamic burden of the aneurysm, it is associated with the risk of aneurysmal rebleeding and regrowth due to retrograde blood flow into the aneurysm [3].

Herein, we present a case of VADA involving the PICA, treated by protecting the PICA with a balloon catheter via the contralateral VA and by performing aneurysmal embolization and proximal PAO from the ipsilateral VA, with a favorable outcome. We also review the related literature.

## Case presentation

**Figure 1:**
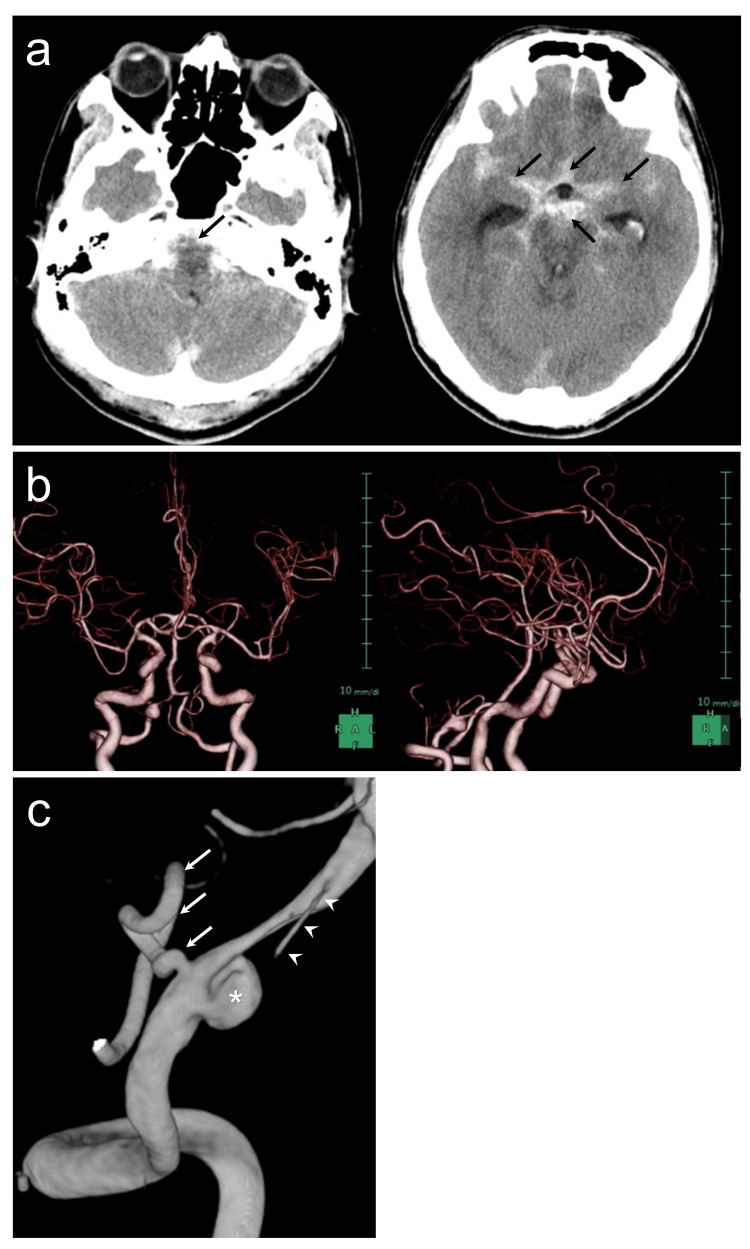
Preprocedural imaging of the case. (a) Preprocedural CT shows subarachnoid hemorrhage in the basal cistern, prepontine cistern, and sylvian fissure (black arrows). (b) Preprocedural CT angiography shows the right vertebral artery dissecting aneurysm (VADA) involving the posterior inferior cerebellar artery (PICA). The left vertebral artery (VA) developed almost equally compared to the right VA. (c) Preprocedural three-dimensional rotational angiography reveals right VADA (white asterisk). The PICA (white arrows) arises from dorsal of the aneurysm, and the anterior spinal artery (ASA, white arrowheads) arises distal to the aneurysm. Stenosis between the aneurysm and the ASA origin is also suspected of dissection.

**Figure 2:**
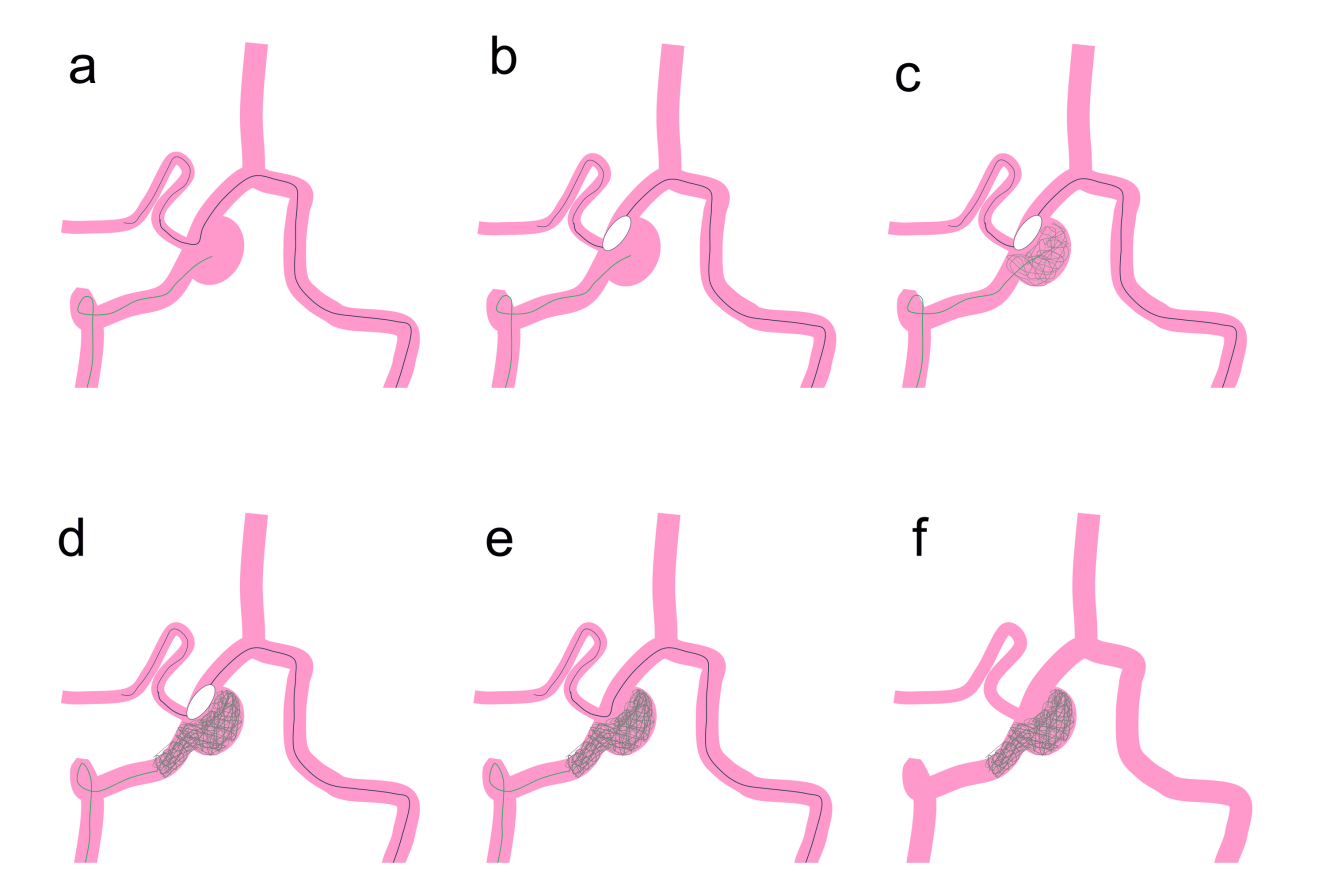
Schema of this technique. (a) First, a balloon catheter over a microguidewire is cannulated to the posterior inferior cerebellar artery (PICA) through the contralateral vertebral artery (VA) and VA union. Second, another microcatheter is inserted into the vertebral artery dissecting aneurysm (VADA) through the ipsilateral VA. (b) Ballon catheter is inflated to protect the PICA. (c) Coil embolization is performed under balloon inflation. (d) Proximal parent artery occlusion is added. (e) Balloon catheter is deflated. (f) Coil embolization of the VADA and proximal VA is achieved. Image credit: Authors

A 60-year-old male with no past history presented to our emergency department with a sudden-onset headache. On arrival, the Glasgow Coma Scale score was 15 (E4V5M6). CT revealed subarachnoid hemorrhage (Figure 1a) and CT angiography indicated dilation of the right VA V4 segment, suggesting a dissecting aneurysm (Figure 1b). Three-dimensional rotational angiography revealed the PICA branching dorsally from the dissection, classified as VADA involving the PICA. ASA originated distally to the dissection, with stenosis between the aneurysm and ASA, also suggesting dissection (Figure 1c). The left VA developed almost equally compared to the right VA. Although preservation of the PICA and ASA was crucial in the endovascular procedure, a simple technique was challenging due to the wide neck aneurysm. The stent-assisted coil embolization can pose the risk of ASA occlusion and early rebleeding caused by stent-related antiplatelet therapy. The proximal PAO can be associated with the risk of aneurysmal rebleeding or regrowth due to retrograde flow into the aneurysm. Therefore, we performed coil embolization of the VADA and proximal PAO via ipsilateral VA under balloon protection of the PICA via the contralateral VA (Figures 2a-2f).

Endovascular Procedure and Postprocedural Course

**Figure 3:**
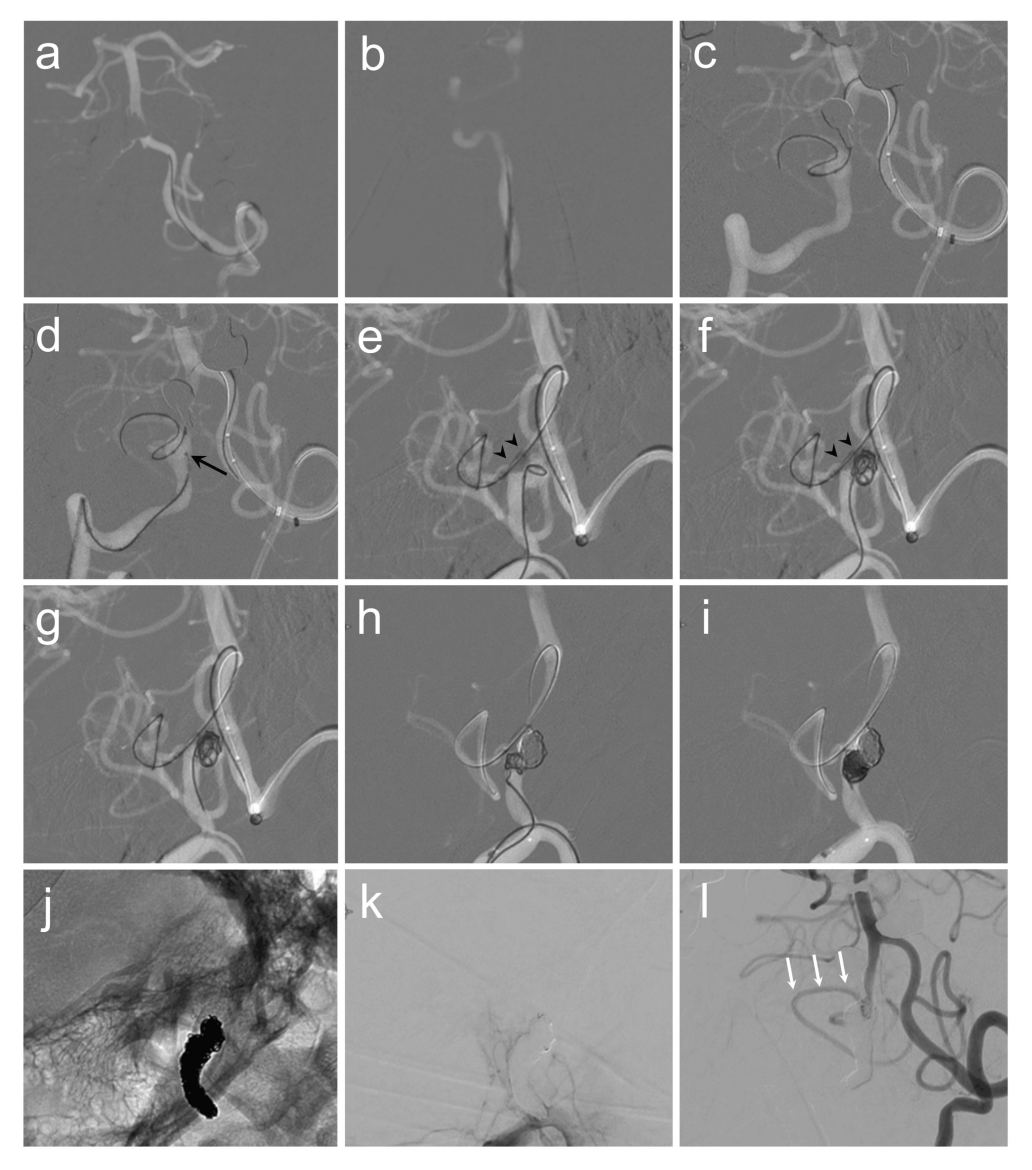
Intraprocedural angiography. (a) A 6F Navien (Medtronic, Irvine, CA) is introduced into the left vertebral artery (VA) V3 segment. (b) A Cerulean DD6 (Medikit, Tokyo, Japan) is introduced to the right VA V2 segment. (c) Under roadmap guidance, a SHORYU HR (Kaneka Medix, Osaka, Japan) over an ASAHI CHIKAI 14 (Asahi Intecc, Aichi, Japan) is cannulated to the right posterior inferior cerebellar artery (PICA) through the VA union via the 6F Navien. (d) A TACTICS (Technocrat, Aichi, Japan) and a Phenom 17 (Medtronic) (black arrow) over an ASAHI CHIKAI 14 are cannulated to the VA V3 segment and vertebral artery dissecting aneurysm (VADA), respectively, via the Cerulean DD6. (e,f) Coil embolization is performed under balloon inflation (black arrowheads). (g) The balloon inflation and deflation are repeatedly performed to confirm stability of the coil mass and prevent ischemic complication of the PICA. (h,i) Proximal parent artery occlusion (PAO) is added after coil embolization of the VADA. (j,k) Embolization of the VADA and proximal PAO is completed. (l) Flow of the PICA is confirmed through the contralateral VA (white arrows).

Antiplatelet therapy was not administered, and only periprocedural heparinization was administered. Under general anesthesia, a 6F guiding sheath (6F Axcelguide, Medikit, Tokyo, Japan) was introduced to the left subclavian artery via transfemoral access. A 6F distal access catheter (6F Navien, Medtronic, Irvine, CA) was navigated to the left VA V3 segment (Figure 3a) through the Axcelguide. A 6F guiding sheath (6F Axcelguide, Medikit) was introduced to the right subclavian artery via transradial access, and a 6F distal access catheter (Cerulean DD6, Medikit) was navigated to the right VA V2 segment (Figure 3b). Using roadmap guidance, a balloon catheter (SHORYU HR, Kaneka Medix, Osaka, Japan) over a microguidewire (ASAHI CHIKAI 14, Asahi Intecc, Aichi, Japan) was delivered to the origin of the right PICA via the 6F Navien through the VA union (Figure 3c). Via the Cerulean DD6, a 3.2F distal access catheter (TACTICS, Technocrat, Tokyo, Japan) and a microcatheter (Phenom 17, Medtronic) over a microguidewire (ASAHI CHIKAI 14) were guided to the right VA V3 segment and aneurysm, respectively (Figure 3d). Aneurysmal coil embolization was performed under balloon inflation to protect the PICA (Figures 3e-3f). Balloon inflation lasted a maximum of three minutes, with a three-minute interval after deflation to confirm the coil stability and prevent ischemic complications of the PICA. Careful attention was paid to avoid extending inflation into the dissected segment (i.e., aneurysm and stenosis distal to the aneurysm) (Figure 3g). The proximal PAO was added to reduce the hemodynamic burden to the dissection, following aneurysmal coiling (Figures 3h-3i). Finally, dense packing of the aneurysm and proximal VA was achieved (Figure 3j). Right VA angiography revealed flow arrest due to coil mass (Figure 3k), and left VA angiography showed the flow of the right PICA through the left VA (Figure 3l).

**Figure 4:**
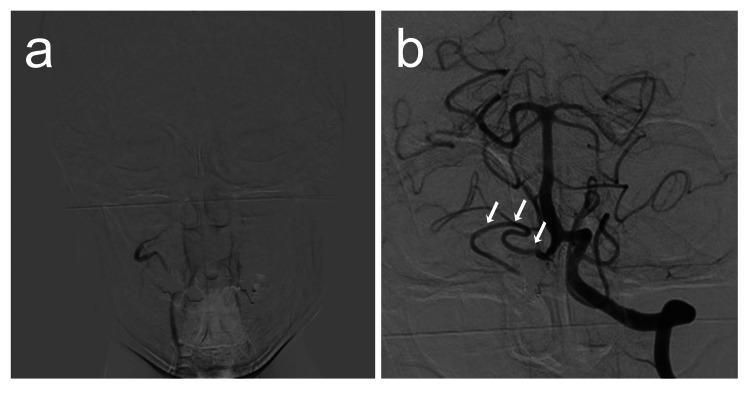
Angiography one year after treatment. (a) Right vertebral artery (VA) angiography shows proximal parent artery occlusion of the VA. Flow in the aneurysm is not confirmed. (b) Left VA angiography shows retrograde flow of the right posterior inferior cerebellar artery (white arrows).

The postprocedural course was uneventful. The patients had not received any antithrombotic medication after the procedure. The patient was discharged with a modified Rankin Scale of 1. Follow-up angiography at one year showed no aneurysmal recurrence or enlargement, with the right PICA flow via contralateral VA (Figures 4a-4b).

## Discussion

The rebleeding rate for untreated VADA was as high as 40.5%, indicating an emergent treatment [4]. Although there is no standardized endovascular procedure for ruptured VADA, it varies depending on the relationship between the dissection and PICA. In cases where the dissection is located distal or proximal to the PICA (VADA distal or proximal to the PICA), internal trapping of the aneurysm is often applied, whereas proximal PAO is usually selected when the dissection involves the PICA (VADA involving the PICA) [5]. However, proximal PAO carries a risk of rebleeding and aneurysmal regrowth due to residual blood flow within the VADA. Table 1 presents a summary of reported cases of endovascular proximal PAO for ruptured VADA involving the PICA [1,6-11]. Including the current case, there were 18 total cases, with an ipsilateral approach in 44.4% (8/18), a contralateral approach in 44.4% (8/18), and a bilateral approach in 11.1% (2/18). The procedure was proximal PAO alone in 83.3% (15/18), and adjunctive techniques include stent-assisted, balloon-assisted, or double catheter techniques in addition to proximal PAO in 16.6% (3/18), which attempt dense packing in the aneurysm. Rebleeding or aneurysmal regrowth due to retrograde flow in the VADA was observed in 27.7% (5/18), which required for additional procedure. In cases with the stent-assisted technique, no rebleeding or aneurysmal regrowth was observed; however, stent use in the acute phase could be associated with the risk of ischemic complications or aneurysmal early rebleeding due to stent use or stent-related antiplatelet therapy [6]. Kitamura et al. suggested the usefulness of the method for proximal PAO from a normal VA to the VADAs just proximal to the PICA origin level with the double catheter technique via contralateral VA [3]. Therefore, the ideal embolization strategy for ruptured VADA involving the PICA suggests the following: (1) preservation of the PICA and ASA by retrograde flow to avoid ischemic complication; (2) tight embolization of the proximal VA and VADA as much as possible to avoid postprocedural aneurysmal regrowth and rebleeding; and (3) no use of the stent to avoid the risk of thrombotic or hemorrhagic complications associated with stent or stent-related antiplatelet therapy.

**Table 1:** Summary of the reported cases of endovascular proximal PAO for a ruptured VADA involving the PICA. Abbreviations: F, female; M, male; mRS, modified Rankin Scale; NA, not available; OA, occipital artery; PAO, parent artery occlusion; PICA, posterior inferior cerebellar artery; VA, vertebral artery; VADA, vertebral artery dissecting aneurysm; WFNS, World Federation of Neurosurgical Societies ^a^ Staged surgery including the endovascular procedure in the acute phase and direct surgery in the chronic phase is planned.

Authors (year)	Age/sex	WFNS grade	Approach	Adjunctive technique	Regrowth or rebleeding	Additional procedure	Follow-up period (months)	Outcome (mRS)
Iihara et al. [7] (2002)	60/M	IV	Ipsilateral	No	Regrowth	Endovascular internal trapping via contralateral VA	20	1
	51/M	V	Ipsilateral	No	Regrowth	OA-PICA anastomosis, PICA origin clip ligation	3	1
Nonaka et al. [8] (2012)	45/F	II	Ipsilateral	No	No	No	2	0
Hamasaki et al. [9](2014)	56/M	V	Ipsilateral	No	No	OA-PICA anastomosis, trapping^a^	5	5
	57/M	V	Ipsilateral	No	NA	OA-PICA anastomosis, trapping^a^	12	1
Ota et al. [1] (2016)	66/M	II	Ipsilateral	No	Regrowth	Stent placement, balloon-assisted coil embolization via contralateral VA	16	NA
	35/F	II	Ipsilateral	No	Regrowth	Stent-assisted coil embolization via contralateral VA	8	NA
Moteki et al. [10] (2017)	41/M	IV	Contralateral	No	No	No	NA	1
	49/F	II	Contralateral	No	No	No	NA	1
	44/M	II	Contralateral	No	No	No	NA	2
	56/M	I	Contralateral	No	No	No	NA	1
	40/M	I	Contralateral	No	No	No	NA	1
	53/M	IV	Contralateral	No	Rebleeding	Coil embolization via contralateral VA	NA	2
	47/M	II	Contralateral	No	No	No	NA	2
Kanematsu et al. [11] (2018)	43/M	NA	Ipsilateral	No	Regrowth	OA-PICA anastomosis, PICA origin clip ligation	168	1
Tsuji et al. [6] (2019)	47/F	III	Bilateral	Stent-assist	No	No	18	1
Kitamura et al. [3] (2024)	43/F	NA	Contralateral	Double-catheter	No	No	14	1
Present case	60/M	I	Bilateral	Balloon-assist	No	No	12	1

Our technique, which uses a balloon catheter instead of a stent, enabled dense packing of the proximal VA and aneurysm while preserving the PICA and reducing the risk of thrombotic or hemorrhagic complications associated with stent or stent-related antiplatelet therapy. Since the purpose of the balloon catheter was to protect the PICA, careful attention was paid to avoid extending inflation into the dissected segment, which may cause vessel occlusion or rupture. The PICA branched dorsally from the aneurysm in this case, which minimized the aforementioned risk. However, depending on the relationship between the aneurysm and PICA origin, this technique may be challenging because balloon catheters facilitate dissection. In addition, this technique requires a bilateral approach and may not be applicable in cases with an undeveloped contralateral VA or steep angulation of the VA union. Further investigation is needed to clarify the usefulness, potential risk, and limitations, including thrombotic events due to the coil itself and coil migration during the procedure.

## Conclusions

This technique allows dense packing while preserving the PICA and avoiding hemorrhagic complications with stent-related antiplatelet therapy. Although further investigation is needed to confirm the potential risk and limitations including thrombotic events due to coil itself and coil migration, it can be a useful therapeutic option for the VADA involving the PICA.
